# The prevalence of delirium in belgian nursing homes: a cross-sectional evaluation

**DOI:** 10.1186/s12877-021-02517-y

**Published:** 2021-11-06

**Authors:** Sabbe Kelly, van Der Mast Roos, Dilles Tinne, Van Rompaey Bart

**Affiliations:** 1 University of Antwerp, Centre for Research and Innovation in Care Belgium, Universiteitsplein 1, 2610 Antwerp, Belgium; 2 Attaché statistician at Statistics Belgium Belgium, 1000 Brussels, Belgium; 3 University of Leiden, Leids Universitair Medisch Centrum The Netherlands, Albinusdreef 2, 2333 ZA Leiden, Netherlands; 4 University of Antwerp, Nurse and Pharmaceutical Care Belgium, Universiteitsplein 1 Sabbe Kelly, MsC, RN 2610 Antwerp, Belgium

**Keywords:** Delirium, delirium point-prevalence, delirium screening, nursing homes, long-term care, precipitating delirium factors

## Abstract

**Background:**

Delirium is a common geriatric syndrome, but only few studies have been done in nursing home residents. Therefore, the aim of this study was to investigate (point) prevalence of and risk factors for delirium in nursing homes in Belgium.

**Methods:**

A multisite, cross-sectional study was conducted in six nursing homes in Belgium.

Residents of six nursing homes were screened for delirium. Exclusion criteria were coma,‘end-of-life’ status and residing in a dementia ward. Delirium was assessed using the Delirium Observation Screening Scale.

**Results:**

338 of the 448 eligible residents were included in this study. Of the 338 residents who were evaluated, 14.2 % (95 %CI:3.94–4.81) screened positive for delirium with the Delirium Observation Screening Scale. The mean age was 84.7 years and 67.5 % were female. Taking antipsychotics (p = 0.009), having dementia (p = 0.005), pneumonia (p = 0.047) or Parkinson’s disease (p = 0.03) were more present in residents with delirium. The residents were more frequently physically restrained (p = 0.001), participated less in activities (p = 0.04), had had more often a fall incident (p = 0.007), had lower levels of cognition (p < 0.001; MoCA ≥ 26, p = 0.04; MoCA ≥ 25, p = 0.008) and a higher “Activities of Daily Living” score (p = 0.001). In multivariable binary logistic regression analysis, a fall incident (2.76; 95 %CI: 1.24–6.14) and cognitive impairment (OR: 0.69; 95 %CI: 0.63–0.77) were significantly associated with delirium.

**Conclusions:**

Delirium is an important clinical problem affecting almost 15 % of the nursing home residents at a given moment. Screening of nursing home residents for risk factors and presence of delirium is important to prevent delirium if possible and to treat underlying causes when present.

## Background

Delirium is a common geriatric syndrome that is characterized by disturbance in attention and awareness. The disturbance develops over a short period of time, represents an acute change from baseline attention and awareness, and tends to fluctuate in severity during the course of a day [1].

The etiology is multifactorial and caused by underlying medical conditions or the use or withdrawal of drugs [4]. Owing to the fluctuating nature and different presentations of the condition, delirium may be difficult to diagnose and is often missed by all healthcare workers [5, 6]. older peopleDelirium basically reflects decompensation of cerebral functions, as a result of one or more pathophysiological processes [3]. Although delirium is encountered in all age groups, older people are considered to be a high-risk group for development of delirium.

Early symptom recognition enables to quickly diagnose and treat the underlying cause(s) and may prevent negative outcomes such as a decline in cognitive and daily functioning, increased morbidity and mortality, and higher health care costs [4, 7–9].

Delirium is a major source of distress for residents, caregivers and health care providers, and has significant societal implications for the resident, the family, the community, and the entire health care system [10–12].

According to health care setting and sample selection criteria, the prevalence of delirium in older people ranges from 9.6 to 89 %, [4]. Residents in long-term care facilities (LTCFs) or nursing homes have a population at a particular higher risk of developing, because predisposing factors such as advanced age, dementia, functional impairment, malnutrition, sensory impairment, and other comorbidities may be prevalent [13, 14].

Only few studies on delirium have been done in nursing homes, with heterogeneous results and prevalences ranging from 1.4 to 70.3 %, where there are no determined percentages for incidence [14–16]. Despite its many adverse effects, delirium is often not adequately recognized by nurses, nurse assistants, doctors or other healthcare personnel during their daily practice in nursing homes. Knowledge on the prevalence of delirium in nursing homes will increase awareness of delirium, not only among nurses but also among other health care professionals and administrators. This might stimulate the implementation of delirium prevention and management in nursing homes if this is deemed necessary.

Therefore, we investigated the (point)prevalence of delirium and associated factors among 338 residents of 65 years and older in six nursing homes.

## Methods

### Study design

This study was a cross-sectional point-prevalence evaluation. Data were collected by three trained research nurses from February 2018 till March 2018 in six nursing homes in Belgium. Out of the 19 contacted nursing homes, six were willing to participate. The nursing homes participating in the study provided long-term health care to residents who were no longer able to care for themselves at home. The nursing homes had nursing permanence 24 h a day, with nurse assistants conducting the main care tasks, under the supervision of a nurse. All residents of a ward of the participating nursing homes were screened at the same time, regardless of their time of admission.

This article adheres to the STROBE checklist for observational research [17].

### Selection criteria

All residents admitted to the participating nursing homes were considered potentially eligible if they were aged 65 years and older, were native Dutch speakers and if they or a proxy provided written informed consent. If there was the (suspicion of) dementia, but the resident resided on a regular ward in the nursing homes, the resident was included. Residents in coma, with aphasia, being in ‘end-of-life” status (residents that were assumed to live only a couple of days) or residents on specific dementia wards were not eligible to participate.

### Procedure

Three research nurses were trained to screen for delirium using an e-learning tool designed to improve healthcare workers’ delirium recognition and knowledge [18, 19].

The nursing homes staff, the residents as well as their families received written information about the study from the research nurses. If requested and/or needed, (additional) oral information was provided. The residents were asked to give an informed consent. If self-determination was impossible, proxy consent was requested from their families. The presence of delirium was assessed in all eligible residents and relevant sociodemographic and clinical data was collected from the residents’ clinical records, taking into account all clinical information of the preceding 24 h before screening. Delirium assessments using the DOSS were done by three trained research nurses. The assessments were performed during the morning or late shift, depending on the availability of the trained nurse researcher and of the residents. All additional information, including residents diagnoses, were collected by conducting a file review of the resident. Being physically restrained was visually determined by the nurse researchers and amounted to any freedom-restricting measure, ranging from a Swedish belt to placing a tray in the seat, which prevented the resident from moving independently.

### Measurement scales

Delirium was evaluated using the 13-tem DOSS [20], a delirium screening scale that is based on the DSM-IV-TR criteria for delirium. The DOSS was especially developed for nurses without specific training in gerontology or behavioural alterations, to improve feasibility of delirium screening in clinical practice and allows bedside use during regular care, repetitively and without respondent burden [21]. A score of 0 is defined as ‘normal behaviour’, meaning absence of behavioural alterations. The highest total score is 13; the cutoff point is ≥ 3. Three or more points indicate that the person is probably delirious and further action is necessary [20]. The pooled estimates of sensitivity (90 %; 76 %-97 %, CI 95 %) and specificity (92 %; 88 %-94 %, CI 95 %) was determined using a hierarchical regression model. The findings indicated a high diagnostic test accuracy for the DOS scale [22].

Cognitive functioning was assessed with the Montreal Cognitive Assessment (MoCA) that is a brief cognitive screening tool with high sensitivity (up to 100 %) and specificity (87 %) for detecting cognitive impairment. The MoCA covers eight cognitive domains including executive functioning, visuoconstructional functioning, attention and concentration, language, memory, conceptual thinking, calculation and orientation. MoCA scores range between 0 and 30. A score of 26 or over is considered to be normal. In a study, people without cognitive impairment scored an average of 27.4; people with mild cognitive impairment (MCI) scored an average of 22.1; people with Alzheimer’s disease scored an average of 16.2 [23]. Dementia was considered present if the patient had a documented diagnosis in the medical record.

The Katz Activities of Daily Living (ADL) was used for assessment of functional status. The Katz was developed as a standardized quantitative measure for evaluating treatment, prognosis and functional changes in patients and residents regardless of their medical condition [24, 25]. In the participating nursing homes, an ordinal rating scale of four categories (from ‘help needed’ to ‘completely independent’) of the six ADL functions bathing, dressing, toileting, transferring, continence and feeding is used [26, 27]. For this study, the scores for the six categories were summarized into one global score, ranging from 4 (completely independent) to 24 (help needed on all ADL functions). The additional criteria of the Katz ADL orientation in time and space were assessed with the MoCA.

Furthermore the use and amount of medication were recorded, as well as the use of feeding tubes, peripheral venous catheters, urinary catheters and physical restraints.

### Statistical analysis

All analyses were conducted witg SPSS® 26.0 (SPSS Inc., Chicago, IL, USA). The normal distribution of the data was assessed Parametric statistics were used to describe the data. Continuous variables were presented with their mean, standard deviation (SD), minimum and maximum, whereas categorical data were presented as the absolute number (n) with percentages.

For the demographic variables and clinical characteristics, comparisons between the two groups (delirium and no delirium) were performed using the Fisher’s exact test, Pearson Chi-Square and Independent t-test. To visualize the interaction between the cognitive functioning, measured with the MoCA and delirium, measured with the DOSS, a scatterplot was used. By univariate logistic regression analysis the association of variables with delirium was evaluated. Variables found to be statistically significant in the univariate analysis were included in a multivariate binary logistic regression model in order to determine the factors associated with delirium and their interactions with each other. The level of significance was established as 95 % (p < 0.05).

### Power analysis

Post-hoc power testing with G*Power showed that based on the study sample size, a power (1 – β) of 89 % for the comparison between the groups and the logistic regression was established [28].

## Results

### Demographic and clinical characteristics of residents with and without delirium

In total, six nursing homes participated. 338 out of 448 eligible residents (75 %) were included. The 110 eligible residents that were not included didn’t give consent or a consent was not given by a proxy. 338 residents were evaluated. The demographic, cognitive, functional and clinical characteristics of residents with and without delirium are presented in Table 1. The mean age of the whole sample was 84.7 years and 67.5 % were female. According to the DOSS, 48 residents 14.2 % scored ≥ 3 and were therefore presumed to have delirium. Residents with delirium were more frequently physically restrained (p = 0.001), participated less in activities (p = 0.04), had had more often a fall incident in the last 90 days (p = 0.007), had lower levels of cognition (p < 0.001; MoCA ≥ 26, p = 0.04; MoCA ≥ 25, p = 0.008) and a higher ADL score (p = 0.001).

**Table 1:** Demographic and clinical characteristics of residents with and without delirium according to the DOSS

Resident characteristics		Entire sample (n = 338), n (%)	Delirium (n = 48, 14.2 %), n (%)	No delirium (n = 290, 85.8 %), n (%)	p-value
Age	(Years) Mean (SD, min. – max.)^1^	84.7 (8.0, 65–102)	85.5 (6.2, 72–97)	84.6 (8.3, 62–102)	0.39^7^
Time being resident	(Years) Mean (SD, min. – max.)^1^	2.7 (2.7, 0–25)	2.5 (2.6, 0–13)	2.7 (2.7, 0–25)	0.72^7^
Gender	Male	110 (32.5)	16 (33.3)	94 (32.4)	0.87^5^
	Female	228 (67.5)	32 (66.7)	196 (67.6)	
Smoking	Yes	34 (10.1)	3 (6.3)	31 (10.7)	0.44^5^
	¬ (Amount) Mean (SD, min. – max.)^1^	1.0 (3.6, 0–25)	0.75 (3.7, 0–24)	1.0 (3.6, 0–25)	0.67^7^
Alcohol use	Yes	98 (29.0)	10 (20.8)	88 (30.3)	0.23^5^
	¬ (Number of drinks) Mean (SD, min. – max.)^1^	0.4 (1.0, 0–10)	1.2 (2.5, 0–7)	1.0 (2.0, 0–7)	0.62^7^
Type of room	Single	260 (76.9)	40 (83.3)	220 (75.9)	0.36^5^
	Double	78 (23.1)	8 (16.7)	70 (24.1)	
Being visited	None	41 (12.1)	9 (18.8)	32 (11.0)	0.30^6^
	Daily	56 (16.6)	4 (8.3)	52 (17.9)	
	Weekly	177 (52.4)	25 (52.1)	152 (52.4)	
	Two-weekly	25 (7.4)	3 (6.3)	22 (7.6)	
	Monthly	39 (11.5)	7 (14.6)	32 (11.0)	
Being physically restrained	Yes	52 (15.4)	16 (33.3)	36 (12.4)	**0.001**^5^
Participating in activities	Never	33.1 (112)	12 (25.0)	100 (34.5)	**0.04**^6^
	Daily	30 (8.9)	1 (2.1)	29 (10.0)	
	Weekly	121 (35.8)	26 (54.2)	95 (32.8)	
	Two-weekly	121 (8.9)	3 (6.3)	27 (9.3)	
	Monthly	45 (13.3)	6 (12.5)	39 (12.4)	
Fall incident last 90 days	Yes	108 (32.0)	24 (50.0)	84 (29.0)	**0.007**^5^
Loss of significant someone	Yes	70 (20.7)	15 (31.3)	55 (19.0)	0.06^5^
Cognitive impairment	(Score) Mean (SD, min. – max.)^1^	16.7 (6.0, 2–30)	9.1 (4.6, 2–19)	18.0 (5.3, 2–30)	**< 0.001**^7^
	MoCA ≥ 26^2^	314 (92.9)	48 (100.0)	266 (91.7)	**0.04**^5^
	MoCA ≥ 25^3^	304 (89.9)	48 (100.0)	256 (88.3)	**0.008**^5^
ADL score^4^	(Score) Mean (SD, min. – max.)^1^	13.4 (4.7, 6–24)	15.6 (4.9, 6–24)	13.1 (4.6, 6–23)	**0.001**^7^
Bladder catheter	Yes	14 (4.1)	3 (6.3)	11 (3.8)	0.4^3^
Medication	(Number) Mean (SD, min. – max.)^1^	8.43 (4.6, 0–29)	8.6 (5.1, 3–25)	8.4 (4.6, 0–29)	0.8^4^
Medication	Antipsychotics	53 (15.7)	14 (29.2)	39 (13.4)	**0.009**^4^
Chronic pathology	Dementia	93 (27.5)	22 (45.8)	71 (24.5)	**0.005**^4^
	Pneumonia	65 (19.2)	4 (8.3)	61 (21.0)	**0.047**^4^
	Parkinson	37 (10.9)	10 (20.8)	27 (9.3)	**0.03**^4^

^1^Mean, SD: Standard Deviation, min: minimum, max: maximum; ^2^Cognitive impairment as determined with the Montreal Cognitive Assessment (MoCA); ^3^Correction for education level; ^4^Activities of Daily Living determined with the Katz; ^5^Fisher’s exact; ^6^Pearson Chi-Square; ^7^Independent t-test

Of all the medication, acute and chronic pathologies analyzed, only taking antipsychotics (p = 0.009), having dementia (p = 0.005), having pneumonia (p = 0.047) and having Parkinson’s disease (p = 0.03) were associated with the presence of delirium. There were no other acute pathologies more present in residents with delirium.

### Factors associated with the presence of delirium

Table 2 shows the factors independently associated with the presence of delirium using univariate and multivariate binary logistic regression analysis. At univariate analysis, taking antipsychotics (p = 0.009), having dementia (p = 0.005), pneumonia (p = 0.047) or Parkinson’s disease (p = 0.03) were more present in residents with delirium. They were more frequently physically restrained (p = 0.001), participated less in activities (p = 0.04), had had more often a fall incident (p = 0.007), had lower levels of cognition (p < 0.001; MoCA ≥ 26, p = 0.04; MoCA ≥ 25, p = 0.008) and a higher ADL score (p = 0.001).

**Table 2:** Factors independently associated with the presence of delirium using univariate and multivariate binary logistic regression analyses

Variable	Category	Univariate analysis			Multivariate analysis		
		**B**	**OR**^**3**^	**95 % CI**^**4**^	**B**	**OR**^**3**^	**95 % CI**^**4**^
Being physically restrained	Yes	1.26	3.52	1.76–7.06			
Participate in activities	Never	Baseline					
	Daily	-0.25	0.78	0.27–2.22			
	Weekly	-1.50	0.22	0.03–1.97			
	Two-weekly	0.58	1.78	0.68–4.66			
	Monthly	-0.33	0.72	0.17–3.14			
Fall incident < 90 days	Yes	0.90	2.45	1.32–4.56	1.21	2.76	1.24–6.14
Cognitive impairment^1^	Score	-0.35	0.70	0.64–0.77	-0.37	0.69	0.63–0.77
ADL score^2^	Score	0.12	1.12	1.05–1.20			
Medication	Antipsychotics	0.98	2.65	1.31–5.38			
Chronic pathology	Dementia	0.96	2.61	1.39–4.89			
	Pneumonia	-1.08	0.34	0.12–0.99			
	Parkinson	0.94	2.56	1.15–5.71			

Dependent variable: Delirium present as determined with the DOSS (yes/no)

^1^Cognitive impairment as determined with the Montreal Cognitive Assessment (MoCA); ^2^Activities of Daily Living determined with the Katz; ^3^Odds Ratio; ^4^95 % Confidence Interval

Nagelkerke R^2^: 59 % (all variables included by “Enter” method); 50 % (as shown in multivariable analysis by “Forward Conditional” method)

In multivariate binary logistic regression analysis, of all associated variables only a fall incident in the last 90 days (2.76; 95 %CI: 1.24–6.14) and cognitive impairment as determined with the MoCA (OR: 0.69; 95 %CI: 0.63–0.77) were associated with delirium. The Nagelkerke R^2^ was 59 % if all variables (significant or not) were included by the “Enter” method and 50 % by using the “Forward Conditional” method where only a fall incident in the last 90 days and cognitive impairment were taken into consideration. This method showed that cognitive impairment and a fall incident the last 90 days were the decisive variables.

### Correlation between cognitive status and delirium

**Fig. 1:**
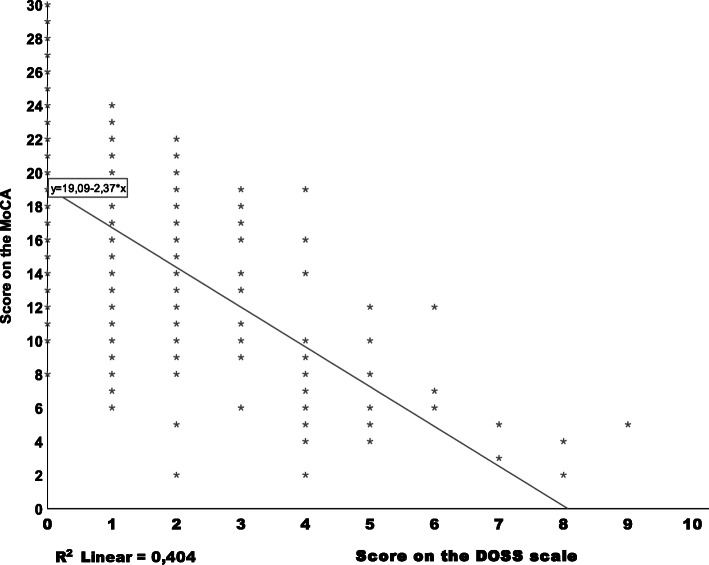
Scatterplot of the Score on the MoCA in relation to the score on the DOSS. Regression equation y = 19.09-2.37x. R^2^ = 0.404

Figure 1 shows the association between the score on the MoCA and the score on the DOSS scale with the regression equation y = 9.09x-2.37.

## Discussion

In this study, delirium point prevalence was determined at more than 14 % of the nursing homes residents. If nursing homeresidents take antipsychotics, have dementia or pneumonia or Parkinson’s disease or had a fall incident the last 90 days they have a significant higher risk in developing delirium. The use of antipsychotics needed to be present for more than 1 week previous to the determination of delirium to be taken into account, to verify they were not prescribed as a response to the delirium development. Furthermore, residents with delirium were more frequently physically restrained, participated less in activities and were more dependable on the health care personnel.

Our prevalence rate of 14.2 % is in in contrast with the results of nursing homes delirium study in Italy reporting a delirium prevalence of 36.8 % [16]. This could be due to the difference in screening tool (Italian study: 4-AT) or the difference in who determined the presence of delirium, which was the attending physician in the Italian study. In Belgium, screening is done by nurses. The attending physician is called upon whendelirium is present [19]. No other differences in methodology were described.

There is a lack of studies that describe the (point)prevalence of delirium [16]. The most influencing factor for the different prevalence estimates in the existing studies is whether or not residents with known dementia are included and the measurement scale used [29]. Studies that include residents with dementia find higher prevalence numbers for the presence of delirium. In the study by Voyer & Coll. [29], residents with a confirmed diagnosis of dementia were excluded, but even then, 89.9 % of the residents included shows cognitive impairment. Studies that included fewer cognitively impaired residents reported delirium prevalence ranging from 6.5 to 21.9 % [30, 31]. This shows that cognitive impairment or dementia and the possible presence of delirium are highly correlated.

The study of Cheung et al. showed an incidence of 40.4 % in Canadian nursing homes between 2010 and 2015, a point prevalence was not determined [14]. Besides these two studies, no recent (< 5 years) studies about delirium in long-term care setting were found upon literature review early 2020. This concludes that delirium has been poorly studied compared to hospital or acute setting [14, 16].

In this study, the association between potentially predisposing and precipitating factors and delirium are similar to those reported in literature [4, 14, 16]. Predisposing conditions such as cognitive impairment and a higher ADL score are common among nursing homeresidents. The precipitating factors, such as fall incidents the last 90 days, the use of physical restraints and medication in general could represent the goal of delirium prevention strategies that are necessary to implement, including fall prevention.the nurse researchers found 15 % of the residents being physically restrained during their observation, which is disturbingly high and an issue that needs to be addressed in further contact with the nursing homes in the light of the adverse effect physical restraints can have on the residents.

The association with antipsychotics might suggest some further considerations; They could have been prescribed for behavioral changes related to dementia but also for behavioral changes related to delirium, since they still represent the most common treatment used in residents with delirium [32]. It cannot be excluded, however, that antipsychotics may have contributed to the promotion of fall incidents or delirium in some patients with cognitive impairment or determined dementia, according to previous studies [32, 33].

In contrast to previous findings, there was no association between benzodiazepine use and delirium [34, 35]. An explanation can be found in the fact that most of the studies on the association between benzodiazepines and delirium were conducted in general hospitals and evaluated the association between the short-term use of benzodiazepines in the context of an acute illness and the occurrence of delirium. In nursing homes, most residents are long-term users of benzodiazepines [34, 35].

This study has some limitations. The gold standard for delirium diagnosis, which is the DSM-5 criteria applied by an expert physician is not possible in the most nursing homes. Screening occurred by trained nurse researchers, because the staff of the nursing homes, mainly consisting of nurse assistants, were not equipped to screen for delirium.

At the same time, the residents were screened only once, which is an important limitation since delirium symptoms come and go during the day. The fluctuating nature of delirium was not taken into consideration to determine the point prevalence, which could have led to an underestimation of delirium presence. Although the residents’ records were analyzed by the trained nurse researchers to look for described sings of delirium, the description of possible symptoms of delirium was often unclear. Nurse assistants, responsible for the residents’ records are not familiar with delirium in Belgium [36, 37].

This study confirms the findings in previous research that delirium is common in nursing homeresidents. They represent a high-risk population for delirium since well-known predisposing and precipitating risk factors are frequently present. Vulnerability for the negative consequences of delirium is high [38]. Therefore, the use of simple and easy to administer screening tools must be implemented in nursing homes.

The high prevalence of delirium in nursing homes implies that all the health care professionals working in this setting must be aware of the relatively high risk of delirium and the risk factors that are associated with delirium among their residents. The screening needs to be done by the staff that is present bedside, in this case the nurse assistants. They need to be trained in recognizing, screening, preventing and treating delirium. Healthcare professionals, especially nurse assistants, that are the main caretakers of residents in nursing homes, should receive delirium training during their basic training, where delirium training is completely lacking at the moment [36, 37]. Even if the minister of education includes delirium in the final attainment levels, there is a gap in knowledge of several years, which we can’t allow. Therefore it is from the utmost importance that nurse assistants and other healthcare personnel working in nursing homes must receive obligated delirium training to be able to recognise, screen for and prevent delirium and prohibit the negative outcomes associated with it [5, 16, 19, 31].

There is a difference in the used measurement scales to asses delirium in nursing homes and who is eligible to perform the screening [2, 14, 16]. Therefore, future research should look into the question who is qualified to screen with what screening tools. The potential adverse consequences of not detecting delirium are far greater than a potential false positive screening, as overlooking its precipitating factors and blaming dementia for any behavioral changes jeopardizes the mental and physical health of residents.

Delirium training can be done by a number of ways, but if the goal is to train all health care personnel, especially nurse assistants that are already working, the training program must be executable on the work floor. A blended learning trajectory would be the ideal way to achieve delirium knowledge as fast and optimal as possible [18, 19]. Future longitudinal studies should assess the prognostic meaning of delirium and the effect of multicomponent prevention and treatment programs in the nursing homesetting [19]. Knowledge on the prevalence of delirium in nursing homes might increase awareness of delirium, not only among nurses but also among other health care professionals and administrators, thus stimulating the implementation of delirium prevention and management in nursing homes.

## Conclusions

Delirium is an important clinical problem affecting almost 15 % of the nursing home residents at a given moment. Nursing home residents represent a high-risk population for delirium since well-known predisposing risk factors are frequently present. Therefore, the use of simple and easy to administer screening tools should be implemented in nursing homes.

Screening of nursing home residents for risk factors and presence of delirium is important to prevent delirium if possible and to treat underlying causes when present.

All health care professionals working in this setting should be aware of the relatively high risk of delirium and the risk factors that associated with delirium among their residents, especially nurse assistants. Healthcare professionals should further receive specific training on delirium.

Knowledge on the prevalence of delirium in nursing homes might increase awareness of delirium, among health care professionals and administrators, thus stimulating the implementation of delirium prevention and management in nursing homes.

## Data Availability

The datasets generated and/or analysed during the current study are not publicly available due due to privacy and ethical restrictions but are available from the corresponding author on reasonable request.

## Abbreviations

ADL: activities of daily living.
CI: confidence interval.
DOSS: delirium observation screening scale.
MoCA: Montreal cognitive assessment.
OR: odds ratio.
SPSS: statistical package for social sciences.
