# Correction to: The prevalence of delirium in Belgian nursing homes: a cross-sectional evaluation

**DOI:** 10.1186/s12877-021-02625-9

**Published:** 2021-12-01

**Authors:** Kelly Sabbe, Roos van der Mast, Tinne Dilles, Bart Van Rompaey

**Affiliations:** 1 University of Antwerp, Centre for Research and Innovation in Care, Universiteitsplein 1, Antwerp, 2610 Belgium; 2 Attaché statistician at Statistics Belgium, Brussels, 1000 Belgium; 3 University of Leiden, Leids Universitair Medisch Centrum, Albinusdreef 2, Leiden, 2333 ZA Netherlands; 4 University of Antwerp, Nurse and Pharmaceutical Care, Universiteitsplein 1, Antwerp, 2610 Belgium

**Correction to: BMC Geriatr 21, 634 (2021)**

**https://doi.org/10.1186/s12877-021-02517-y**

In the original version of this article [1], the given and family names of the authors were incorrectly structured.

The original article [1] has been updated.
